# High-flow oxygen therapy in tracheostomized patients at high risk of weaning failure

**DOI:** 10.1186/s13613-019-0482-2

**Published:** 2019-01-07

**Authors:** Tania Stripoli, Savino Spadaro, Rosa Di mussi, Carlo Alberto Volta, Paolo Trerotoli, Francesca De Carlo, Rachele Iannuzziello, Fabio Sechi, Paola Pierucci, Francesco Staffieri, Francesco Bruno, Luigi Camporota, Salvatore Grasso

**Affiliations:** 1 Dipartimento dell’Emergenza e Trapianti d’Organo (DETO), Sezione di Anestesiologia e Rianimazione, Università degli Studi di Bari “Aldo Moro”, Ospedale Policlinico, Piazza Giulio Cesare 11, Bari, Italy; 2 Dipartimento di Morfologia, Chirurgia e Medicina Sperimentale, Sezione di Anestesiologia e Terapia Intensiva Universitaria, Università degli studi di Ferrara, Ferrara, Italy; 3 Dipartimento di Scienze Biomediche ed Oncologia Umana, Cattedra di Statistica Medica, Università degli Studi Aldo Moro, Bari, Italy; 4 Dipartimento di Medicina Respiratoria e del Sonno, Università degli Studi di Bari “Aldo Moro”, Bari, Italy; 5 Dipartimento dell’Emergenza e Trapianti d’Organo (DETO), Sezione di Chirurgia Veterinaria, Università degli Studi di Bari “Aldo Moro”, Bari, Italy; 6 Department of Adult Critical Care, Guy’s and St Thomas’ NHS Foundation Trust, King’s Health Partners, King’s College London, London, UK; 7 Division of Centre of Human Applied Physiological Sciences, King’s College London, London, UK; 8 Dipartimento di Scienze Chirurgiche e Microchirurgiche, Università degli Studi di Sassari, Sassari, Italy

**Keywords:** High-flow oxygen therapy, Tracheostomy, Weaning from mechanical ventilation, Neuro-ventilatory drive, Work of breathing

## Abstract

**Purpose:**

High-flow oxygen therapy delivered through nasal cannulae improves oxygenation and decreases work of breathing in critically ill patients. Little is known of the physiological effects of high-flow oxygen therapy applied to the tracheostomy cannula (T-HF). In this study, we compared the effects of T-HF or conventional low-flow oxygen therapy (conventional O_2_) on neuro-ventilatory drive, work of breathing, respiratory rate (RR) and gas exchange, in a mixed population of tracheostomized patients at high risk of weaning failure.

**Methods:**

This was a single-center, unblinded, cross-over study on fourteen patients. After disconnection from the ventilator, each patient received two 1-h periods of T-HF (T-HF1 and T-HF2) alternated with 1 h of conventional O_2_. The inspiratory oxygen fraction was titrated to achieve an arterial O_2_ saturation target of 94–98% (88–92% in COPD patients). We recorded neuro-ventilatory drive (electrical diaphragmatic activity, EAdi), work of breathing (inspiratory muscular pressure–time product per breath and per minute, PTP_musc/b_ and PTP_musc/min_, respectively) respiratory rate and arterial blood gases.

**Results:**

The EAdi_peak_ remained unchanged (mean ± SD) in the T-HF1, conventional O_2_ and T-HF2 study periods (8.8 ± 4.3 μV vs 8.9 ± 4.8 μV vs 9.0 ± 4.1 μV, respectively, *p* = 0.99). Similarly, PTP_musc/b_ and PTP_musc/min_, RR and gas exchange remained unchanged.

**Conclusions:**

In tracheostomized patients at high risk of weaning failure from mechanical ventilation, T-HF did not improve neuro-ventilatory drive, work of breathing, respiratory rate and gas exchange compared with conventional O_2_ after disconnection from the ventilator. The present findings might suggest that physiological effects of high-flow therapy through tracheostomy substantially differ from nasal high flow.

## Introduction

High-flow nasal cannula oxygen therapy is the administration of a warmed and humidified air/oxygen mixture at a flow rate between 20 and 60 L/min [1] through nasal cannulae (HFNC). HFNC is increasingly used in several clinical contexts, particularly in de novo hypoxemic respiratory failure [2–5]; post-extubation [6, 7] and in post-cardiothoracic surgery [8]. Compared with conventional oxygen therapy (conventional O_2_), HFNC produces several physiological effects [9], which include: a better matching between the delivered gas flow mixture and patient’s spontaneous inspiratory flow; a positive end-expiratory pressure (PEEP) effect (generally between 2 and 8 cm H_2_O [10, 11]) and a “CO_2_ wash out” effect from the upper airways [12, 13]. Further, the humidified and warmed gas mixture favors the mucociliary function and reduces upper airway resistance [2, 14]. The synergistic combination of these mechanisms leads to improved oxygenation [2], decrease in neuro-ventilatory drive and work of breathing [15–17].

High-flow oxygen therapy can also be applied to the tracheostomy opening (T-HF) in tracheostomized patients. Since HFNC decreases the risk of re-intubation in patients at risk of extubation failure [6, 7], it is reasonable to hypothesize that T-HF could aid the separation from mechanical ventilation in mechanically ventilated, tracheostomized patients at high risk of weaning failure. However, T-HF is a novel therapy and it is not known whether it replicates the physiological advantages of HFNC.

Our study hypothesis was that T-HF, compared to conventional O_2_, would decrease the neuro-ventilatory drive, work of breathing, respiratory rate and improve gas exchange, in a mixed population of tracheostomized patients at high risk of weaning failure [18].

## Methods

### Study design

This was a single-center, unblinded, cross-over study of ventilated patients via tracheostomy with prolonged weaning from mechanical ventilation. After disconnection from the ventilator, each patient received two 1-h periods of T-HF (T-HF1 and T-HF2) alternated with 1 h of conventional O_2_.

### Patient selection

Patients admitted to the intensive care unit (ICU) of the Bari University Hospital (Italy) between June 2017 and May 2018, requiring mechanical ventilation via tracheostomy, were screened for inclusion in the study. Inclusion criteria were age ≥ 18 and a diagnosis of prolonged weaning according to WIND study (i.e., patients not weaned after more than 7 days from the first separation attempt) [18]. Exclusion criteria were: contraindications to the insertion of an electrical activity of the diaphragm (EAdi) catheter (e.g., upper gastrointestinal surgery, esophageal varices, esophageal trauma) cardiopulmonary instability, concomitant neuro-muscular pathologies and/or known phrenic nerve dysfunction. Furthermore, we excluded patients showing signs of paradoxical abdominal movement or use of accessory inspiratory muscles. The local ethics committee approved the study protocol (Azienda Ospedaliero-Universitaria Policlinico di Bari Ethics Committee, Protocol No.: 55276/C.E. Lug 2016). Patients or next of kin gave their written consent to participate in the study.

Based on our clinical protocol [19], patients were disconnected from the ventilator and allowed to breath unassisted through the tracheostomy cannula if they met the following criteria: (a) resolution or improvement in the condition leading to acute respiratory failure; (b) positive end-expiratory pressure (PEEP) lower than 5 cm H_2_O and an inspiratory oxygen fraction (FiO_2_) lower than 0.5 with a PaO_2_/FiO_2_ ratio greater than 150 mm Hg; (c) arterial pH > 7.35; Richmond Agitation-Sedation Scale (RASS) between 0 and − 1 [20], on no sedation or on a continuous infusion of dexmedetomidine (0.1–1.4 μg/kg/h); (d) ability to trigger the ventilator as demonstrated by a decrease in pressure airway opening (*P*_AO_) > 3–4 cm H_2_O during a brief (5–10 s) end-expiratory occlusion test. Other criteria were hemodynamic stability without vasoactive drugs (excluding a dobutamine and/or dopamine infusion lower than 5 μg/kg/min and a 3 μg/kg/min, respectively) and normothermia.

Patients were ventilated with a Servo-i ventilator (Maquet, Getinge group Critical Care, Solna, Sweden) equipped with the EAdi software (Maquet, Getinge group Critical Care, Solna, Sweden). The EAdi catheter was positioned based on the corrected nose–earlobe–xyphoid distance formula, and its position was subsequently adjusted using the ventilator EAdi catheter position tool (Servo-i ventilator NAVA software) in accordance with the manufacturer’s instructions [21].

### Measurements

Patients were studied in the semi-recumbent position. The EAdi signal was collected from the RS232 port of the ventilator at a sampling rate of 100 Hz (NAVA tracker software, Getinge Critical Care, Solna, Sweden) and stored in a personal computer. The NAVA tracker files were subsequently converted and analyzed using the ICU Lab software package (Kleistek Engineering; Bari, Italy).

The inspiratory EAdi peak (EAdi_PEAK_), the integral of the inspiratory EAdi deflection over time (EAdi_PTP_), the slope of the EAdi from the beginning of inspiration to the peak (EAdi_SLOPE_), the respiratory rate (RR) and the neural inspiratory time (Ti_NEUR_) were measured from the EAdi trace [22].

The pressure generated by the inspiratory muscles (Pmusc) was estimated from the EAdi signal and the neuro-muscular efficiency index (NME), through to a method previously validated by Bellani and coworkers [23]. The NME is the ratio between the negative pressure generated by the inspiratory muscles and the corresponding EAdi [17, 24–26] and is therefore expression of the efficiency of the diaphragmatic depolarization (as expressed by the EAdi) to generate a muscular contraction.

Accordingly, Pmusc may be estimated through the formula:

$${\text{Pmusc}} = {\text{EAdi}}*{\text{NME}}$$

In our study, we obtained the NME by the ratio between the negative pressure in the airway opening (*P*_AO_) generated by a single inspiratory effort against the occluded airways and the corresponding positive peak in EAdi (EAdi_PEAK_). Briefly, we performed a short (5–10 s) end-expiratory occlusion and recorded the *P*_AO_ and EAdi traces that were subsequently digitally recorded and analyzed offline. We analyzed a single inspiratory effort, the first that occurred during the end-expiratory airway occlusion. Of note, this method allows calculating the NME without an esophageal catheter, since the fall in *P*_AO_ during a spontaneous inspiratory effort against the occluded airways is, by definition, equal to the corresponding fall in esophageal pressure (P_ES_) [27, 28]. However, the *P*_AO_-based NME overestimates by a factor of 1.25 the P_ES_-based NME, and therefore we corrected our Pmusc calculation as follows [23]:

$${\text{Pmusc}} = {\text{EAdi}}*\left( {{\text{NME}}/1.25} \right)$$

The inspiratory Pmusc pressure–time product per breath (PTP_musc/b_) was calculated as the area under the Pmusc signal. The inspiratory Pmusc pressure–time product per minute (PTP_musc/min_) was calculated as:

$${\text{PTP}}_{\text{musc/min}} = {\text{PTP}}_{\text{musc/b}} *{\text{RR}}.$$

The tidal volume (VT) was not measured to avoid any modification in breathing pattern caused by the measurement apparatus.

### Study protocol

Before disconnection from the ventilator, each patient underwent a 30-min spontaneous breathing trial (SBT) in pressure support ventilation (PSV), with a pressure support of 5 cm H_2_O and a PEEP of 5 cm H_2_O [29]. The SBT was successful if no signs of distress occurred, defined as: (a) paradoxical abdominal movements or other signs of accessory respiratory muscle fatigue; (b) cardiovascular instability (systolic blood pressure, SBP > 160 or < 90 mmHg—or a 20% change from the pre-SBT values; heart rate, HR > 120 or < 60 beats/min or 20% change from the pre-SBT values; (c) arterial desaturation with SaO_2_ < 88%; (d) hypercapnia defined as respiratory acidosis with pH < 7.35 [19]. Patients succeeding the SBT were separated by the ventilator and included in the study.

The NME was calculated during the SBT, 5–10 min before the disconnection from mechanical ventilation, after careful tracheal suctioning in order to improve the quality of the measurement.

Weaning from the ventilator was defined as the ability to breathe spontaneously through the tracheostomy cannula without signs of respiratory distress (see above) and without the need for reconnection to the mechanical ventilator for at least 48 h.

**Fig. 1:**
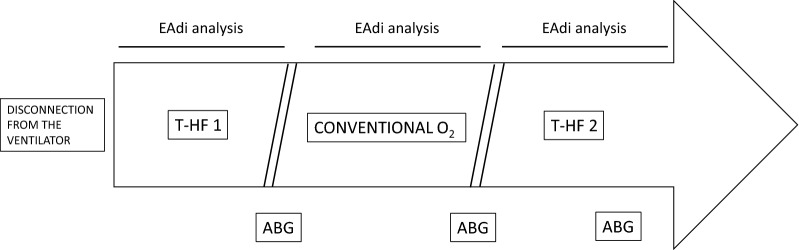
Study protocol timeline. ABG, arterial blood gas analysis; EAdi, diaphragm electrical activity; T-HF 1, first period of high-flow tracheostomy cannula oxygen therapy (1 h); conventional O2, period of conventional low-flow oxygen therapy (1 h); TP, T-piece; T-HF 2, second period of high-flow tracheostomy cannula oxygen therapy (1 h)

Immediately after disconnection from the ventilator, patients underwent a cross-over protocol with an ON–OFF–ON design, alternating T-HF with conventional O_2_ delivered through a T-piece (i.e., T-HF1—conventional O2 therapy—T-HF2), with each phase lasting for 1 h (Fig. 1).

**Fig. 2:**
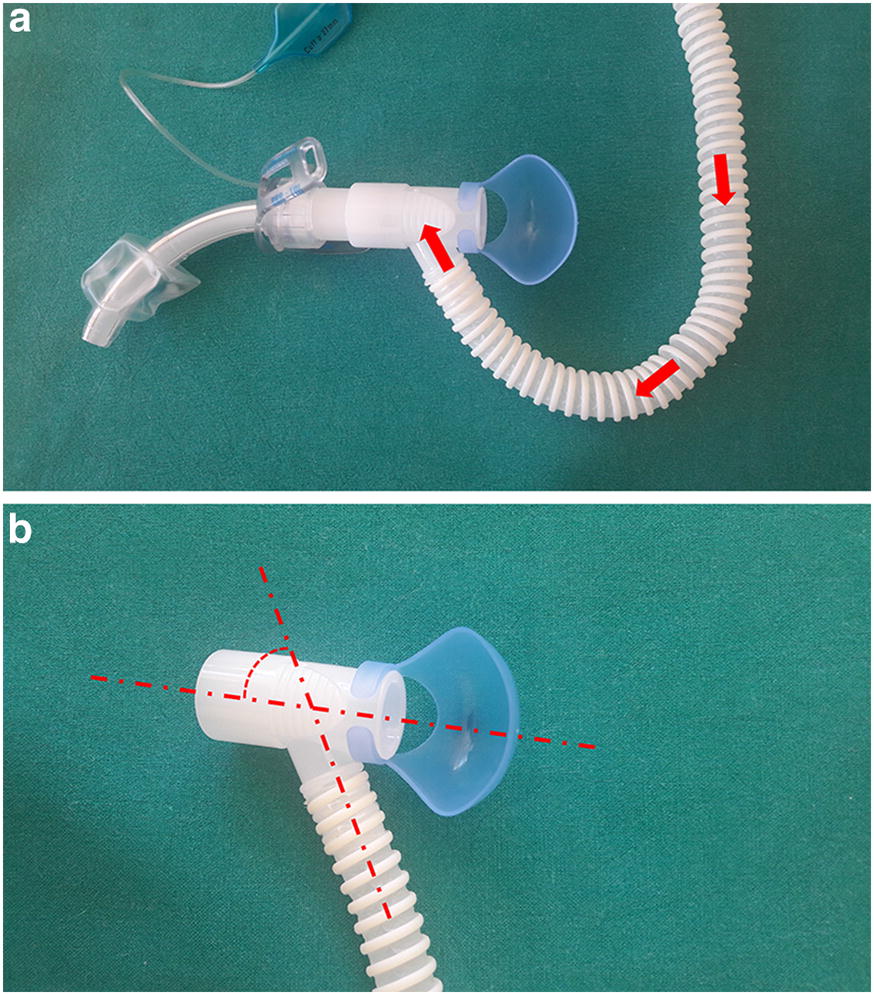
Gas flows through the specific interface for the tracheostomy tube (OPT870, Fisher and Paykel, Healthcare, Auckland, New Zealand) tested in the present study. The interface (**a**) is composed of a connector (length = 38 mm) equipped with a side stream gas delivery tube (diameter 12 mm). The angle between the axes of the connector and the delivery tube is 60° (**b**)

The T-HF was administered through the AIRVO™ 2 system (Fisher and Paykel Healthcare, Auckland, New Zealand) with a specific interface for the tracheostomy tube (OPT870, Fisher and Paykel Healthcare, Auckland, New Zealand). The system allows administration of humidified and warmed gas flow at 10–60 L/min in the adult configuration. The interface (Fig. 2) is composed of a connector (length = 38 mm) equipped with a side stream gas delivery tube (diameter 12 mm). The angle between the axis of the connector and the delivery tube is 60°. The gas flow was set at 50 L/min in all the patients. At the beginning of each T-HF period, the FiO_2_ was titrated to achieve an arterial hemoglobin oxygen saturation (SaO_2_) of 94–98% (or 88–92% in patients with chronic obstructive pulmonary disease). The temperature of the heater–humidifier was set at 37 °C.

Conventional O_2_ therapy was administered through a T-piece weaning kit connected to a standard O_2_/air mixer (0–15 L/min). The T-piece gas flow was set to 10 L/min in all the patients. At the beginning of the conventional O_2_ period, the FiO_2_ was titrated to match the same oxygenation targets of the T-HF periods (see above).

At the end of each study period, arterial blood for gas analysis was obtained. The EAdi trace was digitally recorded and analyzed for the entire duration of the study period.

### Statistical analysis

The power analysis indicated a sample size of 14 patients taking the EAdi_PEAK_ as the main study endpoint. We have hypothesized that the variability explained by the study condition had to be at least 5% of total variability that approximatively corresponds to an expected effect size of 0.25, with a power of 0.8 and a significance level of 0.05. The sample size was determined using the software GPower version 3.1.9.2.

Continuous quantitative variables were summarized as mean ± standard deviation if normally distributed or as median and interquartile range if non-normally distributed. Comparisons were performed with ANOVA for repeated measures or Friedman test as appropriate. A *p* value < 0.05 was considered as statistically significant, except in multiple comparison procedure, when the p-value was adjusted. The analyses were carried out with SAS 9.4 for Windows PC.

## Results

**Fig. 3:**
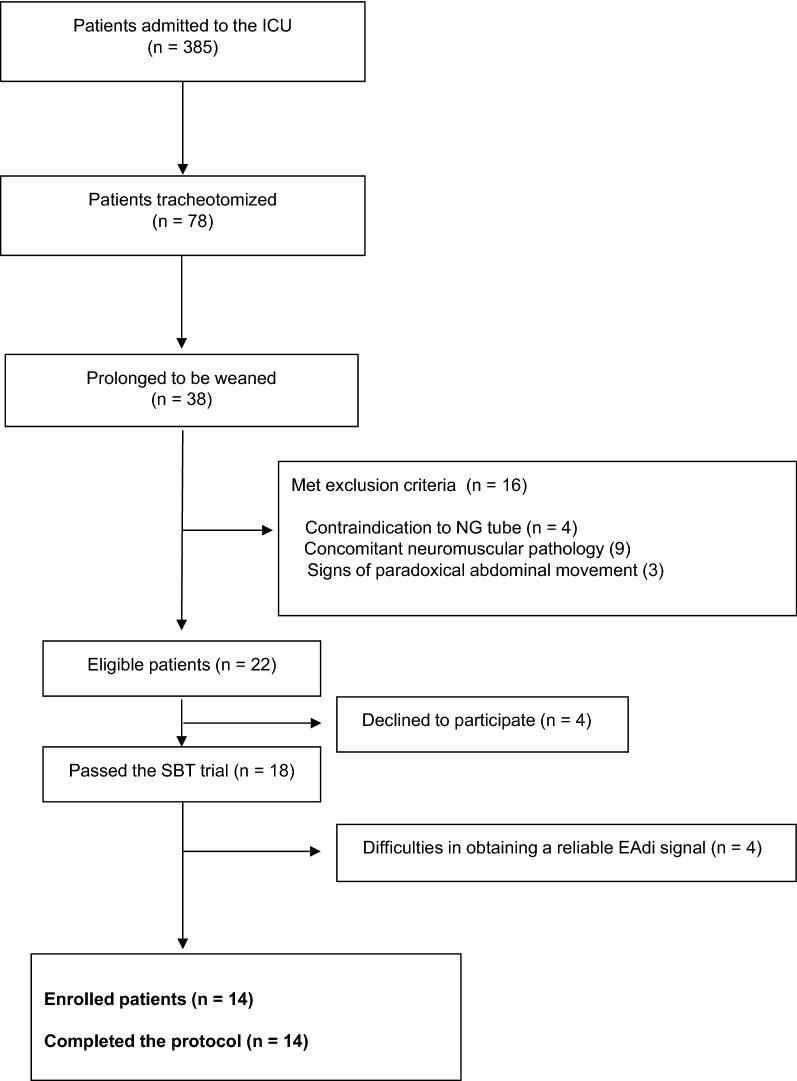
Flow diagram of patient’s enrollment. Abbreviation: EAdi, diaphragm electrical activity; ICU, intensive care unit; NG, nasogastric tube

The CONSORT study diagram (Fig. 3) shows that in the study period, 22 out of 38 tracheostomized patients with prolonged weaning from mechanical ventilation at high risk of weaning failure were eligible for the study and 14 were finally enrolled and completed the study protocol. Patients’ demographics and clinical characteristics are shown in Table 1.

**Table 1:** Demographical and clinical characteristics of studied patients

Patient	Gender	Age	SAPS II (at ICU admission)	SOFA (day of study)	Reason of admission in ICU	Days of MV	Disconnection outcome	ICU length of stay (days)	ICU outcome
1	F	71	40	2	Polytrauma	11	Failure	17	Survivor
2	M	66	41	3	Thoracic trauma	14	Success	18	Survivor
3	F	73	59	4	Acute coronary syndrome	14	Success	20	Survivor
4	M	72	55	3	Post-anoxic coma	25	Failure	55	Non-survivor
5	M	58	29	5	Subarachnoid hemorrhage	11	Success	15	Survivor
6	F	49	27	4	Postoperative respiratory failure (neurosurgery)	25	Failure	45	Non-survivor
7	F	74	44	2	Cardiac failure	15	Failure	25	Non-survivor
8	M	41	30	2	Community-acquired pneumonia	21	Failure	30	Survivor
9	M	71	43	4	COPD exacerbation	16	Failure	28	Survivor
10	M	38	24	5	Polytrauma	12	Failure	28	Survivor
11	M	52	72	2	Septic shock (urinary tract infection)	10	Failure	20	Survivor
12	M	80	56	7	COPD exacerbation	21	Failure	28	Survivor
13	M	57	26	5	Subarachnoid hemorrhage	15	Success	20	Survivor
14	M	48	25	3	Polytrauma	13	Success	18	Survivor

### Breathing pattern and gas exchange

Table 2 shows the breathing pattern and gas exchange parameters. The oxygenation target was achieved with similar FiO_2_ levels in the three study steps (0.51 ± 0.1; 0.51 ± 0.12; and 0.51 ± 0.11 in the T-HF1, conventional O_2_ and T-HF2 periods, respectively; *p* = NS). The arterial pO_2_ was 109 ± 27 mm Hg in the T-HF1 period, 92 ± 17 mm Hg in the conventional O_2_ period and 111 ± 28 mm Hg in the T-HF2 period, *p* = 0.09. Arterial PCO_2_, RR and Ti_NEUR_, remained similar throughout the study (Table 2).

**Table 2:** Breathing pattern and gas exchange in different experimental conditions

	HF-T 1	Conventional O_2_	HF-T 2	*p*
RR (breaths/min)	19.4 ± 3.9	20.1 ± 4.3	20.4 ± 5.4	0.94
Ti_NEUR_ (s)	1.07 ± 0.21	1.03 ± 0.2	1.02 ± 0.24	0.88
pH	7.51 ± 0.04	7.51 ± 0.04	7.52 ± 0.04	0.76
PaCO_2_ (mm Hg)	44 ± 9	44 ± 8	43 ± 8	0.93
HCO_3_^−^ (mEq/L)	33 ± 4.5	33 ± 4	33 ± 4	0.99
PaO_2_ (mm Hg)	109 ± 27	92 ± 17	111 ± 28	0.09
PaO_2_/FiO_2_	219 ± 59	187 ± 50	223 ± 45	0.13

Data are expressed as mean ± standard deviation

HF-T, high-flow tracheotomy cannula oxygen therapy; conventional O_2_, conventional low-flow oxygen therapy through a T-piece; RR, respiratory rate; Ti_NEUR_, neural inspiratory time; PaCO_2_, arterial partial carbon dioxide pressure; PaO_2_, arterial partial oxygen pressure; FiO_2_, inspiratory oxygen fraction

### Neuro-ventilatory drive and work of breathing

**Fig. 4:**
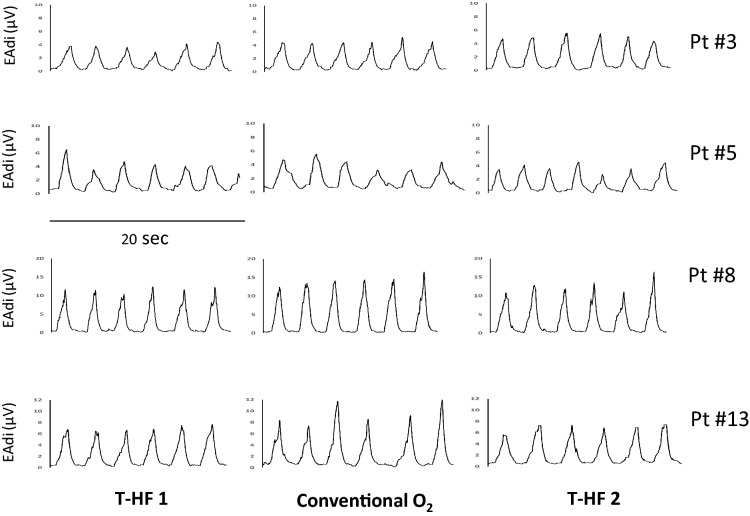
Experimental record showing the diaphragm electrical activity (EAdi) in the three experimental conditions in four representative patients. T-HF 1, first period of high-flow tracheostomy cannula oxygen therapy; conventional O2, period of conventional low-flow oxygen therapy with T-piece; T-HF 2, second period of high-flow tracheostomy cannula oxygen therapy

**Fig. 5:**
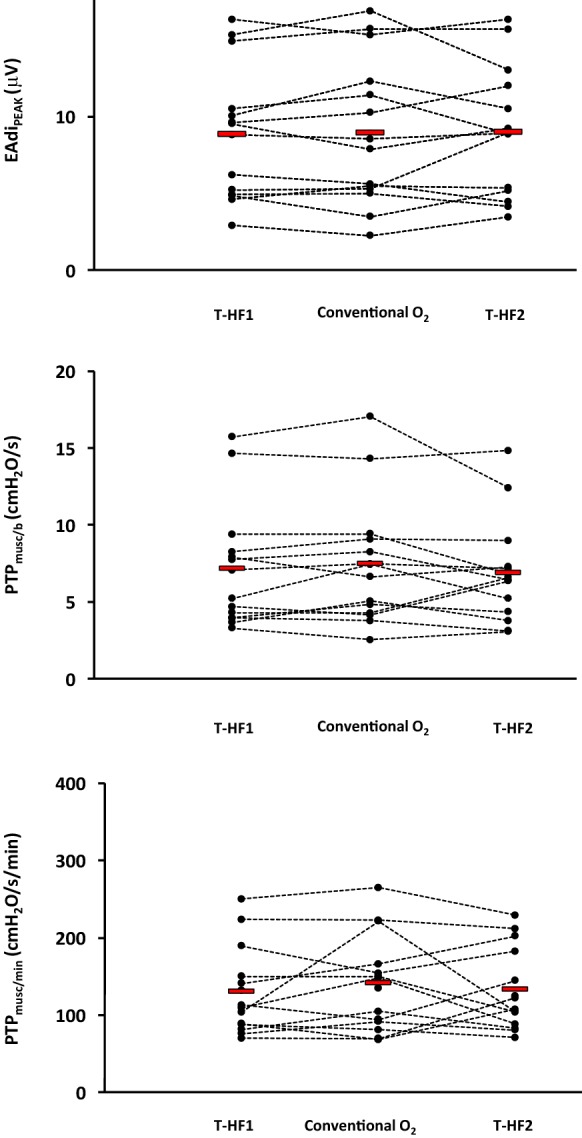
Trend of the neuro-ventilatory drive, as expressed by the diaphragm electrical activity peak EAdi_PEAK_, and of work of breathing, as expressed by the inspiratory muscular pressure–time product per breath (PTP_musc/b_) and per minute (PTP_musc/min_). Other abbreviations: T-HF 1, first period of high-flow tracheostomy cannula oxygen therapy; conventional O2, period of conventional low-flow oxygen therapy with T-piece; T-HF 2, second period of high-flow tracheostomy cannula oxygen therapy

Figure 4 is an experimental record showing the EAdi traces obtained at the end of each of the three experimental conditions, in four representative patients. The neuro-ventilatory drive, as expressed by the EAdi_PEAK_, EAdi_PTP_ and EAdi_SLOPE_, remained unchanged in the three experimental periods. The same occurred for work of breathing, as expressed by PTP_musc/b_ and PTP_musc/min_ (Table 3). Figure 5 shows the individual changes in EAdi_PEAK_ and work of breathing parameters, in the three experimental conditions.

**Table 3:** Neuro-ventilatory drive and work of breathing parameters

	HF-T 1	Conventional O_2_	HF-T 2 P
EAdi_PEAK_ (μV)	8.8 ± 4.3	8.9 ± 4.8	9 ± 4.2 0.99
EAdi_PTP_ (μV/s)	7.1 ± 3.6	7.8 ± 4.5	6.9 ± 3.2 0.92
EAdi_SLOPE_	8.5 ± 4.3	8.7 ± 5.2	9.1 ± 4.4 0.95
PTP_musc/b_ (cm H_2_O/s)	7.1 ± 3.9	7.4 ± 4.1	6.9 ± 3.4 0.92
PTP_musc/min_ (cm H_2_O/s/min)	130 ± 56	140.7 ± 61	132 ± 53 0.86

Data are expressed as mean ± standard deviation if normally distributed or as median and interquartile range if non-normally distributed

HF-T, high-flow tracheotomy cannula oxygen therapy; conventional O_2_, conventional low-flow oxygen therapy through a T-piece; EAdi_PEAK_, diaphragm electrical activity peak; EAdi_PTP_, EAdi deflection inspiratory area; EAdi_SLOPE_, EAdi slope from the beginning of inspiration to EAdi_PEAK_; PTP_musc/b_, inspiratory pressure–time product per breath; PTP_musc/min_, inspiratory pressure–time product per minute

## Discussion

In this study, we tested the effects of high-flow oxygen therapy through a dedicated tracheostomy interface on gas exchange, neuro-ventilatory drive, respiratory rate and work of breathing in tracheostomized patients at high risk of weaning failure. Our data suggest that T-HF does not produce the same physiological effects as HFNC on these outcome parameters. Although our study was not designed for a detailed examination of the physiological mechanisms generated by high flow at the tracheal level, we discuss the possible physiological mechanisms that may explain our results.

Recent studies have shown that during HFNC, patients decrease their minute inspiratory volume maintaining stable the PaCO_2_ [15–17]. These findings indirectly support the hypothesis that HFNC decreases the neuro-ventilatory drive and work of breathing mainly through its “CO_2_ wash out” effect [11, 13, 15–17]. Since we found that T-HF does not impact on the neuro-ventilatory drive and work of breathing, we speculate that these negative results are explained by the fact that T-HF has negligible “CO_2_ wash out effect” compared with HFNC. This can be explained by the fact that HFNC washes out the CO_2_ mainly from the nasopharyngeal anatomical dead space [12, 13], whereas the tracheostomy tube bypasses the upper airways. The effect of tracheostomy on dead space has been confirmed by Chadda et al. [30] who show that physiological dead space increases from 156 ± 67 mL before decannulation to 230 ± 82 ml after decannulation.

We were not able to show any impact of T-HF on the PaO_2_/FiO_2_ ratio, compared with conventional O_2_ (Table 2). We have no data on tracheal pressures and lung volumes, but—given that conventional O2 and T-HF achieved similar results on a multitude of parameters—it is unlikely that a “PEEP effect” occurred in our patients. Our results are in contrast with those reported in a research letter by Corley et al. [31], showing a slight, but statistically significant improvement in oxygenation with T-HF compared to conventional O_2_ in prolonged wean patients. Corley et al. applied T-HF for 15 min, whereas our study periods lasted 1 h and the gas delivery flow rate during conventional O_2_ was 15 L/min versus 10 L/min in our study. However, it seems unlikely that these methodological differences could explain the discrepancy between Corley’s results and ours.

The EAdi is a “processed” diaphragmatic electromyography signal recorded through an array of electrode pairs mounted on the wall of a nasogastric feeding tube [32] and is proportional to the intensity of the electrical stimuli directed to the diaphragm [24, 33, 34]. In other words, the EAdi is a measure of the discharge rate of phrenic nerves that have been shown to reliably reflect the neuro-ventilatory drive and work of breathing [24, 32–36]. Recently, Bellani and coworkers [23] demonstrated that EAdi can be used to estimate the instantaneous work of breathing, as we did in our study. Liu and coworkers found that an EAdi_PEAK_ lower than 15–20 μV during a spontaneous breathing trial was associated with extubation success [25]. Dres et al. [37] and Barwing et al. [38] obtained similar results. In our study, we found that the EAdiP_EAK_ was below 20 μV (Fig. 5) both during the T-HF and the conventional O_2_ periods. We speculate that these findings could be explained by the fact that our patients were tracheostomized. Previous studies have shown that the neuro-ventilatory drive is up to 30% lower in tracheostomized patients [30], probably because the tracheostomy decreases the overall airway resistance [39] and the physiological dead space [30]. Furthermore, to our knowledge, data on the normal ranges of neuro-ventilatory drive in tracheostomized patients are scanty. In analogy with EAdi, the PTP_musc/min_, a well-known index of work of breathing, was within its “acceptable” range (i.e., between 50 and 150 cm H_2_O/s/min [40, 41]), in 72%, 65% and 72% of our patients in the T-HF1, conventional O_2_ and T-HF2 periods, respectively (Fig. 5).

We must acknowledge some study limitations. *First,* we conducted a non-randomized ON–OFF–ON cross-over study that, for practical reasons, could not be blinded. Furthermore, we were not able to measure several respiratory parameters (VT, EELV, airways pressure) that could have provided us with a more complete interpretation of the treatment effect. However, our study was conducted in spontaneously breathing patients and we sought to avoid any modification in breathing pattern caused by the measurement apparatus. *Second,* we estimated Pmusc based on a method recently validated by Bellani and coworkers [22] and applied in different studies, but we must point out that this method assumes a diaphragm contribution to the overall Pmusc of 75%, as occurs in normal conditions [42]. On the other hand, if the contraction of the accessory inspiratory muscles is dominant (as occurs in case of paradoxical abdominal movements) the estimation of Pmusc from EAdi could be biased. However, we excluded from our study patients showing signs of paradoxical abdominal movement and use of accessory inspiratory muscles (see “Methods” section). In addition, the method described by Bellani et al. [23] assumes a linear relationship between EAdi and Pmusc at different lung volumes but other authors have shown a nonlinearity between EAdi and lung volumes [24]. However, this occurs only in case of intense diaphragmatic contractions [24]. *Fourth,* our physiological study was not designed to test the impact of T-HF on patients’ comfort. Over a prolonged period, however, T-HF could better preserve the mucociliary function compared with conventional O_2_ [14], improving the secretion clearance and patient’s comfort. Further studies are needed to test this hypothesis. *Fifth,* we used a fixed high flow rate (50 L/min) but it is possible that, in analogy with HFNC [43], our results would have been different at different flow rates. Further studies are needed to construct a dose–response between T-HF flow rate and physiological effects. On the other hand, a different interface design, for example, with a different flow delivery angle (Fig. 2), could improve the CO_2_ clearing from the tracheal space, compared with the interface tested in the present study.

## Conclusions

In conclusion, in our cohort of tracheostomized patients at high risk of weaning failure, after disconnection from the ventilator T-HF did not affect neuro-ventilatory drive, work of breathing, respiratory rate and gas exchange, compared to conventional O_2_. The present findings might suggest that physiological effects of high-flow therapy through tracheostomy substantially differ from nasal high flow.

## Abbreviations

COPD: chronic obstructive pulmonary disease
Conventional O_2_: conventional low-flow oxygen therapy
EAdi: electric activity of the diaphragm
FiO_2_: inspiratory oxygen fraction
HFNC: high-flow nasal cannula oxygen therapy
ICU: intensive care unit
NIV: noninvasive ventilation
NME: neuro-muscular efficiency
*P*_AO_: pressure airway opening
PEEP: positive end-expiratory pressure
PEEPi: intrinsic positive end-expiratory pressure
PTP_musc/b_: pressure–time product per breath
PTP_Dmuscmin_: pressure–time product per minute
RR: respiratory rate
SaO_2_: arterial hemoglobin oxygen saturation
SBP: systolic blood pressure
SBT: Spontaneous Breathing Trial
T-HF: high-flow tracheostomy oxygen therapy
Ti_NEUR_: neural inspiratory time
VT: tidal volume
